# Supplementary material for: Drivers of species knowledge across the tree of life
Source: eLife. 2023 Oct 17;12:RP88251. doi: 10.7554/eLife.88251 (PMC10581686; doi:10.7554/eLife.88251)
Supplement: Supplementary file 1. — (a) Estimated regression parameters for the full models. (b) Estimated regression parameters for the subset models modeling the number of papers in the Web of Science. (c) Estimated regression parameters for the subset models modeling the number of views in Wikipedia. [file elife-88251-supp1.docx]

**Supplementary File 1a. Estimated regression parameters for the full models modeling the influence of species-level traits and cultural factors on scientific (Web of Science) and societal interest (Wikipedia) for different taxa, as well as the relative scientific and societal interest (Residuals).** CI: 95% Confidence interval.

| **Model** | **Type** | **Parameter** | **Beta** | **SE** | **CI_low** | **CI_high** | **z** | **p** |
| --- | --- | --- | --- | --- | --- | --- | --- | --- |
| Web of Science | - | Intercept | 0.889 | 0.492 | -0.075 | 1.853 | 1.808 | 0.071 |
| Web of Science | Species trait | Organism size | 0.445 | 0.111 | 0.228 | 0.663 | 4.012 | <0.001 |
| Web of Science | Species trait | Colorful [yes] | 0.217 | 0.145 | -0.068 | 0.502 | 1.493 | 0.136 |
| Web of Science | Species trait | Range size | 0.598 | 0.057 | 0.486 | 0.71 | 10.481 | <0.001 |
| Web of Science | Species trait | Domain [freshwater] | 0.251 | 0.315 | -0.366 | 0.868 | 0.797 | 0.426 |
| Web of Science | Species trait | Domain [marine] | -0.27 | 0.279 | -0.817 | 0.276 | -0.969 | 0.332 |
| Web of Science | Species trait | Domain [terrestrial] | -0.546 | 0.216 | -0.97 | -0.122 | -2.525 | 0.012 |
| Web of Science | Species trait | Taxonomic uniqueness (Family) | -0.408 | 0.077 | -0.559 | -0.257 | -5.306 | <0.001 |
| Web of Science | Cultural trait | IUCN [threatened] | 1.229 | 0.192 | 0.853 | 1.604 | 6.41 | <0.001 |
| Web of Science | Cultural trait | IUCN [non-threatened] | 0.974 | 0.154 | 0.672 | 1.277 | 6.315 | <0.001 |
| Web of Science | Cultural trait | Common name [yes] | 0.968 | 0.165 | 0.645 | 1.291 | 5.874 | <0.001 |
| Web of Science | Cultural trait | Human use [yes] | 1.026 | 0.121 | 0.788 | 1.264 | 8.456 | <0.001 |
| Web of Science | Cultural trait | Harmful to humans [yes] | 1.732 | 0.243 | 1.257 | 2.208 | 7.139 | <0.001 |
| Web of Science | Cultural trait | Phylogenetic distance to humans | -0.73 | 0.382 | -1.479 | 0.02 | -1.907 | 0.056 |
| Wikipedia | - | Intercept | 5.973 | 0.354 | 5.28 | 6.666 | 16.891 | <0.001 |
| Wikipedia | Species trait | Organism size | 0.844 | 0.096 | 0.657 | 1.032 | 8.841 | <0.001 |
| Wikipedia | Species trait | Colorful [yes] | 0.744 | 0.125 | 0.498 | 0.99 | 5.932 | <0.001 |
| Wikipedia | Species trait | Range size | 0.214 | 0.037 | 0.14 | 0.287 | 5.701 | <0.001 |
| Wikipedia | Species trait | Domain [freshwater] | 0.827 | 0.291 | 0.256 | 1.398 | 2.838 | 0.005 |
| Wikipedia | Species trait | Domain [marine] | 0.3 | 0.287 | -0.261 | 0.862 | 1.048 | 0.295 |
| Wikipedia | Species trait | Domain [terrestrial] | 0.248 | 0.214 | -0.172 | 0.668 | 1.157 | 0.247 |
| Wikipedia | Species trait | Taxonomic uniqueness (Family) | -0.416 | 0.067 | -0.548 | -0.284 | -6.186 | <0.001 |
| Wikipedia | Cultural trait | IUCN [threatened] | 0.841 | 0.162 | 0.522 | 1.159 | 5.176 | <0.001 |
| Wikipedia | Cultural trait | IUCN [non-threatened] | 0.303 | 0.126 | 0.055 | 0.55 | 2.397 | 0.017 |
| Wikipedia | Cultural trait | Common name [yes] | 1.491 | 0.15 | 1.196 | 1.785 | 9.919 | <0.001 |
| Wikipedia | Cultural trait | Human use [yes] | 0.985 | 0.109 | 0.771 | 1.199 | 9.028 | <0.001 |
| Wikipedia | Cultural trait | Harmful to humans [yes] | 1.827 | 0.242 | 1.354 | 2.301 | 7.56 | <0.001 |
| Wikipedia | Cultural trait | Phylogenetic distance to humans | -0.957 | 0.287 | -1.519 | -0.395 | -3.337 | 0.001 |
| Residuals | - | Intercept | -2.291 | 0.41 | -3.095 | -1.487 | -5.584 | <0.001 |
| Residuals | Species trait | Organism size | 0.276 | 0.103 | 0.074 | 0.478 | 2.684 | 0.007 |
| Residuals | Species trait | Colorful [yes] | 0.426 | 0.133 | 0.165 | 0.688 | 3.197 | 0.001 |
| Residuals | Species trait | Range size | -0.162 | 0.046 | -0.253 | -0.072 | -3.527 | <0.001 |
| Residuals | Species trait | Domain [freshwater] | 0.042 | 0.332 | -0.607 | 0.692 | 0.128 | 0.898 |
| Residuals | Species trait | Domain [marine] | 0.637 | 0.309 | 0.031 | 1.243 | 2.06 | 0.039 |
| Residuals | Species trait | Domain [terrestrial] | 0.464 | 0.223 | 0.028 | 0.9 | 2.084 | 0.037 |
| Residuals | Species trait | Taxonomic uniqueness (Family) | -0.187 | 0.071 | -0.327 | -0.047 | -2.614 | 0.009 |
| Residuals | Cultural trait | IUCN [threatened] | -0.179 | 0.195 | -0.561 | 0.204 | -0.915 | 0.36 |
| Residuals | Cultural trait | IUCN [non-threatened] | -0.334 | 0.153 | -0.634 | -0.033 | -2.179 | 0.029 |
| Residuals | Cultural trait | Common name [yes] | 0.71 | 0.155 | 0.406 | 1.013 | 4.584 | <0.001 |
| Residuals | Cultural trait | Human use [yes] | -0.312 | 0.129 | -0.565 | -0.059 | -2.418 | 0.016 |
| Residuals | Cultural trait | Harmful to humans [yes] | -0.32 | 0.269 | -0.847 | 0.207 | -1.189 | 0.234 |
| Residuals | Cultural trait | Phylogenetic distance to humans | -0.475 | 0.304 | -1.072 | 0.121 | -1.561 | 0.118 |

**Supplementary File 1b. Estimated regression parameters for the subset models modeling the influence of species-level traits and cultural factors on scientific interest (N° opf papers in the Web of Science) for Chordata, Arthropoda and Tracheophyta.** CI: 95% Confidence interval; NA: Not Available.

| **Model** | **Type** | **Parameter** | **Beta** | **SE** | **CI_low** | **CI_high** | **z** | **p** |
| --- | --- | --- | --- | --- | --- | --- | --- | --- |
| Chordata | Intercept | Intercept | -0.144 | 0.649 | -1.415 | 1.128 | -0.221 | 0.825 |
| Chordata | Species trait | Organism size | 1.423 | 0.207 | 1.016 | 1.829 | 6.858 | <0.001 |
| Chordata | Species trait | Colorful [yes] | 0.238 | 0.205 | -0.163 | 0.639 | 1.163 | 0.245 |
| Chordata | Species trait | Range size | 0.818 | 0.085 | 0.65 | 0.985 | 9.567 | <0.001 |
| Chordata | Species trait | Domain [freshwater] | 0.315 | 0.451 | -0.57 | 1.199 | 0.697 | 0.486 |
| Chordata | Species trait | Domain [marine] | -0.797 | 0.387 | -1.556 | -0.038 | -2.057 | 0.04 |
| Chordata | Species trait | Domain [terrestrial] | -0.398 | 0.223 | -0.834 | 0.039 | -1.786 | 0.074 |
| Chordata | Cultural trait | IUCN [threatened] | 1.176 | 0.202 | 0.781 | 1.571 | 5.834 | <0.001 |
| Chordata | Cultural trait | IUCN [non-threatened] | 0.891 | 0.166 | 0.565 | 1.216 | 5.363 | <0.001 |
| Chordata | Species trait | Taxonomic uniqueness (Genus) | -0.17 | 0.086 | -0.338 | -0.001 | -1.969 | 0.049 |
| Chordata | Cultural trait | Common name [yes] | 0.773 | 0.48 | -0.168 | 1.713 | 1.61 | 0.107 |
| Chordata | Cultural trait | Human use [yes] | 0.501 | 0.132 | 0.242 | 0.76 | 3.796 | <0.001 |
| Chordata | Cultural trait | Harmful to humans [yes] | 1.247 | 0.347 | 0.568 | 1.927 | 3.598 | <0.001 |
| Arthropoda | Intercept | Intercept | -0.308 | 0.97 | -2.209 | 1.592 | -0.318 | 0.75 |
| Arthropoda | Species trait | Organism size | -0.403 | 0.247 | -0.888 | 0.082 | -1.628 | 0.103 |
| Arthropoda | Species trait | Colorful [yes] | 0.439 | 0.308 | -0.165 | 1.043 | 1.423 | 0.155 |
| Arthropoda | Species trait | Range size | 0.429 | 0.111 | 0.212 | 0.646 | 3.878 | <0.001 |
| Arthropoda | Species trait | Domain [freshwater] | -0.378 | 0.978 | -2.294 | 1.539 | -0.386 | 0.699 |
| Arthropoda | Species trait | Domain [marine] | -0.48 | 0.933 | -2.308 | 1.349 | -0.514 | 0.607 |
| Arthropoda | Species trait | Domain [terrestrial] | -0.588 | 0.889 | -2.331 | 1.154 | -0.662 | 0.508 |
| Arthropoda | Cultural trait | IUCN [threatened] | 1.828 | 1.188 | -0.501 | 4.157 | 1.539 | 0.124 |
| Arthropoda | Cultural trait | IUCN [non-threatened] | -1.02 | 0.637 | -2.27 | 0.229 | -1.6 | 0.109 |
| Arthropoda | Species trait | Taxonomic uniqueness (Genus) | -0.132 | 0.109 | -0.345 | 0.082 | -1.208 | 0.227 |
| Arthropoda | Cultural trait | Common name [yes] | 0.904 | 0.27 | 0.375 | 1.433 | 3.349 | 0.001 |
| Arthropoda | Cultural trait | Human use [yes] | 3.334 | 0.646 | 2.068 | 4.6 | 5.163 | <0.001 |
| Arthropoda | Cultural trait | Harmful to humans [yes] | 2.065 | 0.442 | 1.199 | 2.932 | 4.671 | <0.001 |
| Tracheophyta | Intercept | Intercept | 0.387 | 2.114 | -3.756 | 4.53 | 0.183 | 0.855 |
| Tracheophyta | Species trait | Organism size | 0.422 | 0.285 | -0.136 | 0.981 | 1.481 | 0.139 |
| Tracheophyta | Species trait | Colorful [yes] | 0.401 | 0.374 | -0.331 | 1.133 | 1.074 | 0.283 |
| Tracheophyta | Species trait | Range size | 0.968 | 0.279 | 0.421 | 1.514 | 3.471 | 0.001 |
| Tracheophyta | Species trait | Domain [freshwater] | NA | NA | NA | NA | NA | NA |
| Tracheophyta | Species trait | Domain [marine] | NA | NA | NA | NA | NA | NA |
| Tracheophyta | Species trait | Domain [terrestrial] | -1.786 | 1.793 | -5.301 | 1.728 | -0.996 | 0.319 |
| Tracheophyta | Cultural trait | IUCN [threatened] | 2.05 | 0.902 | 0.283 | 3.817 | 2.274 | 0.023 |
| Tracheophyta | Cultural trait | IUCN [non-threatened] | 1.694 | 0.558 | 0.601 | 2.788 | 3.037 | 0.002 |
| Tracheophyta | Species trait | Taxonomic uniqueness (Genus) | -0.172 | 0.15 | -0.466 | 0.121 | -1.15 | 0.25 |
| Tracheophyta | Cultural trait | Common name [yes] | 1.078 | 0.48 | 0.137 | 2.019 | 2.245 | 0.025 |
| Tracheophyta | Cultural trait | Human use [yes] | 1.047 | 0.426 | 0.211 | 1.882 | 2.455 | 0.014 |
| Tracheophyta | Cultural trait | Harmful to humans [yes] | 0.849 | 0.829 | -0.775 | 2.473 | 1.025 | 0.305 |

**Supplementary File 1c. Estimated regression parameters for the subset models modeling the influence of species-level traits and cultural factors on popular interest (views in Wikipedia) for Chordata, Arthropoda and Tracheophyta.** CI: 95% Confidence interval; NA: Not Available.

| **Model** | **Type** | **Parameter** | **Beta** | **SE** | **CI_low** | **CI_high** | **z** | **p** |
| --- | --- | --- | --- | --- | --- | --- | --- | --- |
| Chordata | Intercept | Intercept | 7.523 | 0.701 | 6.149 | 8.898 | 10.73 | <0.001 |
| Chordata | Species trait | Organism size | 1.555 | 0.171 | 1.22 | 1.891 | 9.098 | <0.001 |
| Chordata | Species trait | Colorful [yes] | 0.446 | 0.18 | 0.093 | 0.798 | 2.479 | 0.013 |
| Chordata | Species trait | Range size | 0.446 | 0.055 | 0.339 | 0.554 | 8.118 | <0.001 |
| Chordata | Species trait | Domain [freshwater] | 2.107 | 0.393 | 1.337 | 2.877 | 5.362 | <0.001 |
| Chordata | Species trait | Domain [marine] | 0.398 | 0.412 | -0.41 | 1.207 | 0.966 | 0.334 |
| Chordata | Species trait | Domain [terrestrial] | 0.091 | 0.203 | -0.306 | 0.488 | 0.448 | 0.654 |
| Chordata | Cultural trait | IUCN [threatened] | 0.539 | 0.16 | 0.225 | 0.853 | 3.364 | 0.001 |
| Chordata | Cultural trait | IUCN [non-threatened] | -0.011 | 0.131 | -0.268 | 0.246 | -0.086 | 0.931 |
| Chordata | Species trait | Taxonomic uniqueness (Genus) | -0.261 | 0.07 | -0.398 | -0.124 | -3.742 | <0.001 |
| Chordata | Cultural trait | Common name [yes] | 0.623 | 0.34 | -0.043 | 1.29 | 1.832 | 0.067 |
| Chordata | Cultural trait | Human use [yes] | 0.645 | 0.111 | 0.428 | 0.862 | 5.826 | <0.001 |
| Chordata | Cultural trait | Harmful to humans [yes] | 1.205 | 0.328 | 0.563 | 1.847 | 3.678 | <0.001 |
| Arthropoda | Intercept | Intercept | 6.06 | 0.808 | 4.476 | 7.645 | 7.497 | <0.001 |
| Arthropoda | Species trait | Organism size | 0.857 | 0.214 | 0.437 | 1.277 | 4.002 | <0.001 |
| Arthropoda | Species trait | Colorful [yes] | 0.754 | 0.232 | 0.299 | 1.209 | 3.249 | 0.001 |
| Arthropoda | Species trait | Range size | 0.099 | 0.071 | -0.04 | 0.238 | 1.396 | 0.163 |
| Arthropoda | Species trait | Domain [freshwater] | -0.254 | 0.76 | -1.744 | 1.236 | -0.334 | 0.738 |
| Arthropoda | Species trait | Domain [marine] | -0.23 | 0.761 | -1.721 | 1.261 | -0.302 | 0.762 |
| Arthropoda | Species trait | Domain [terrestrial] | -0.009 | 0.679 | -1.34 | 1.322 | -0.013 | 0.99 |
| Arthropoda | Cultural trait | IUCN [threatened] | 1.81 | 0.995 | -0.139 | 3.76 | 1.82 | 0.069 |
| Arthropoda | Cultural trait | IUCN [non-threatened] | -0.32 | 0.571 | -1.44 | 0.8 | -0.56 | 0.575 |
| Arthropoda | Species trait | Taxonomic uniqueness (Genus) | -0.149 | 0.073 | -0.293 | -0.006 | -2.04 | 0.041 |
| Arthropoda | Cultural trait | Common name [yes] | 1.2 | 0.205 | 0.797 | 1.602 | 5.847 | <0.001 |
| Arthropoda | Cultural trait | Human use [yes] | 3.021 | 0.631 | 1.784 | 4.259 | 4.785 | <0.001 |
| Arthropoda | Cultural trait | Harmful to humans [yes] | 2.357 | 0.384 | 1.605 | 3.109 | 6.141 | <0.001 |
| Tracheophyta | Intercept | Intercept | 4.479 | 1.328 | 1.876 | 7.082 | 3.373 | 0.001 |
| Tracheophyta | Species trait | Organism size | 0.268 | 0.187 | -0.098 | 0.634 | 1.436 | 0.151 |
| Tracheophyta | Species trait | Colorful [yes] | 0.495 | 0.244 | 0.017 | 0.974 | 2.029 | 0.042 |
| Tracheophyta | Species trait | Range size | 0.48 | 0.129 | 0.226 | 0.733 | 3.714 | <0.001 |
| Tracheophyta | Species trait | Domain [terrestrial] | NA | NA | NA | NA | NA | NA |
| Tracheophyta | Species trait | IUCN [threatened] | NA | NA | NA | NA | NA | NA |
| Tracheophyta | Species trait | Domain [terrestrial] | 1.363 | 0.921 | -0.442 | 3.168 | 1.48 | 0.139 |
| Tracheophyta | Cultural trait | IUCN [threatened] | 2.218 | 0.558 | 1.124 | 3.313 | 3.975 | <0.001 |
| Tracheophyta | Cultural trait | IUCN [non-threatened] | 1.633 | 0.433 | 0.785 | 2.482 | 3.772 | <0.001 |
| Tracheophyta | Species trait | Taxonomic uniqueness (Genus) | -0.02 | 0.114 | -0.244 | 0.204 | -0.177 | 0.859 |
| Tracheophyta | Cultural trait | Common name [yes] | 1.37 | 0.326 | 0.731 | 2.008 | 4.203 | <0.001 |
| Tracheophyta | Cultural trait | Human use [yes] | 0.985 | 0.281 | 0.435 | 1.535 | 3.508 | <0.001 |
| Tracheophyta | Cultural trait | Harmful to humans [yes] | -0.169 | 0.549 | -1.244 | 0.906 | -0.308 | 0.758 |
